# Feasibility of a multimodal AI-based clinical assessment platform in emergency care: an exploratory pilot study

**DOI:** 10.3389/fdgth.2025.1657583

**Published:** 2025-10-03

**Authors:** Philipp Russ, Peter M. Mross, Gunter Kräling, Fabian Lechner, Mohamed Eldakar, Nadine Schlicker, Simon Bedenbender, Sylvain Brouwer, Kirsten Zantvoort, Andreas Jerrentrup, Ivica Grgic, Martin C. Hirsch

**Affiliations:** ^1^ Institute for Artificial Intelligence in Medicine, Marburg University, University Hospital Giessen and Marburg, Marburg, Germany; ^2^ Department of Internal Medicine and Nephrology, Marburg University, University Hospital Giessen and Marburg, Marburg, Germany; ^3^ Department of Emergency Medicine, Marburg University, University Hospital Giessen and Marburg, Marburg, Germany

**Keywords:** artificial intelligence (AI), clinician decision support, digital health, emergency departments (ED), overcrowding, triage

## Abstract

**Background:**

Overcrowding in emergency departments (EDs) is a key challenge in modern healthcare, affecting not only patient and staff comfort but also mortality rates and quality of care. Artificial intelligence (AI) offers the potential to optimize ED workflows by automating processes such as triage, history-taking and documentation. To explore a potential approach to overcrowding, we developed a multimodal and modular AI-based platform that integrates these functions into a single system. This exploratory pilot study investigated the feasibility of implementing the platform, focusing particularly on usability and patient trust in the system.

**Methods:**

Ambulatory patients triaged as non-urgent at the Marburg University Hospital ED were recruited. After providing written consent, they underwent an AI-supported initial assessment, including vital sign monitoring, automated triage, suspected diagnosis and automatic report generation. Participants then completed validated questionnaires on usability, Trust in Automation (TiA), and a supplementary self-developed survey.

**Results:**

A total of 20 patients were enrolled (70% female, 30% male; mean age 45.1 years), with an average interaction time of 10.6 min. The majority (80%) reported feeling safe, satisfied, and willing to recommend the system, while areas for improvement were identified regarding patient inclusion in decision-making and the perceived quality of information received. Usability was rated as excellent, with a mean System Usability Scale (SUS) score of 90.6. Although familiarity with the system was low, trust-related measures assessed using the TiA questionnaire were generally high.

**Conclusion:**

This exploratory pilot study demonstrates the feasibility and user acceptance of a multimodal AI platform in an ED setting. The system achieved high patient satisfaction, excellent usability, and a generally high level of trust. While these findings are limited to feasibility and perception, they indicate that such systems could serve as a basis for multicenter studies that directly evaluate impacts on triage accuracy, patient engagement, and clinical efficiency.

## 1 Introduction

Overcrowding in emergency departments (EDs) represents one of the most pressing systemic challenges in modern healthcare systems (1). Its consequences extend far beyond patient discomfort, adversely affecting mortality rates, quality of care, and the wellbeing of healthcare professionals (2–4). In response to this multifaceted issue, we developed a modular artificial intelligence (AI)-based prototype platform designed to support key stages of ED workflows. The system is designed to help mitigate overcrowding by assisting with triage, documentation, and clinical decision-making. In the following, we first contextualize our approach by outlining the structural causes of ED overcrowding and then explore how AI technologies may help address them.

ED overcrowding is commonly categorized into three domains that significantly affect patient flow: input, throughput and output factors (5). Input-related issues concern ED access, including high patient volumes, long wait times, and severe or complex presentations (6). Limited access to primary care contributes to rising ED use, particularly for non-emergency conditions (7). Throughput factors refer to internal ED processes, ranging from patient admission to clinical decision-making. These include diagnostic burden, delays in test results, and administrative tasks such as shift structures and staffing shortages (6, 8). Output-related barriers concern patient discharge or transfer, including limited inpatient bed capacity, transport delays, and lack of follow-up care (2, 9).

Recent studies suggest that all three factors capturing the key aspects of ED overcrowding are worsening: Input-related stressors are increasing due to population ageing and increased multimorbidity (10), declining access to primary care providers (11), and an increase in ED visits related to climate-sensitive health issues (12). Throughput challenges are driven by persistent shortages of medical staff and increasing workplace strain (13, 14). Output capacity is increasingly constrained by declining inpatient bed capacity across many regions (15).

In response to these challenges, AI has emerged as a promising tool to optimize clinical workflows, reduce staff burden, and support decision-making in emergency care (16, 17). Beyond its established applications in fields such as radiology and dermatology (18, 19), generative AI tools based on large language models (LLMs) can transcribe clinical conversations in real time and assist in documenting patient histories (20). Furthermore, AI and machine learning (ML) are already being used for triage in EDs.

Recent systematic and narrative reviews synthesize the current state of evidence on AI in EDs and ED triage. They consistently report that demographic characteristics, vital signs, and unstructured free-text data are the most commonly used predictor variables, and the integration of clinical text has been shown to further improve discriminatory performance (21–23). Overall, these reviews suggest that AI approaches, particularly those leveraging natural language processing (NLP) or LLMs, hold promise for enhancing triage and diagnostic accuracy, reducing variability in decision-making and supporting more consistent patient assessment (21, 24–27). At the same time, they underline persistent feasibility challenges, including integration with electronic health records (EHR), issues of interpretability, and the need to secure clinician acceptance (21, 28). While AI models already demonstrate good predictive accuracy for outcomes such as hospital admission and disposition, prospective validation in real-world ED settings remain scarce (29, 30). To move the field forward, several reviews explicitly call for multicenter prospective studies with transparent reporting, seamless EHR integration, and the inclusion of operational outcome metrics to determine the real-world impact of AI-assisted triage (21, 26, 28).

Building on these insights and the identified gaps, we designed a multimodal AI-based platform intended to support multiple stages of ED care. In line with the input, throughput, and output framework, input factors might be addressed through targeted patient management and shorter waiting times; throughput could benefit from reduced clinician workload via streamlined documentation and predictive diagnostics; and output factors may be improved through resource planning enabled by predictions of length of stay. While these potential benefits remain to be demonstrated empirically, they illustrate how AI could, in principle, support ED care beyond isolated tracks.

Our multimodal AI-based platform integrates these functionalities into a single access interface for non-urgently triaged patients who would otherwise remain in the waiting area. Accessible via a self-service cabin, the system conducts history-taking, captures vital signs, performs triage, and supports clinical decision-making by suggesting diagnoses, generating reports, and offering recommendations. Given the critical nature of ED environments and the novelty of such systems especially in high-stakes settings such as emergency medical care, it is crucial to understand how such AI systems are perceived by users, before their potential operational impact can be systematically evaluated. Therefore, this exploratory pilot study aims to evaluate patient perceptions of the platform, with particular emphasis on its usability and perceived trustworthiness.

## 2 Methods

### 2.1 Structure of the multimodal AI-based platform

The multimodal AI-based platform was developed to support the initial assessment of patients in the ED. It consists of a compact unit incorporating a patient monitor, medical sensors, a camera, a microphone, speakers, a screen and intelligent software components, including the diagnostic tool Ada (Ada Health GmbH, Berlin, Germany) (31), an AI-powered speech recorder, and a large language model (LLM). To protect sensitive data, we employed the locally running LLM Mistral Small 3 (mistral-small:24b) developed by Mistral AI, Paris, France (32). The model card is provided in Supplementary Table S1.

Once the patient is connected to the system by medical staff, the assessment begins through a combination of automated data collection and dialog-based history-taking. A virtual avatar guides the patient through a structured interview using Ada to collect symptom information. Simultaneously, vital signs such as heart rate, respiratory rate, oxygen saturation, body temperature, and optionally blood pressure (via a standard cuff) are recorded. The structured symptom data collected by Ada, along with the recorded vital signs, are then fed as input into the LLM.

After the dialogue concludes, all data are aggregated and processed by the backend. The system then generates a category from a five-level triage scale, suggests a suspected diagnosis, and produces a medical report including clinical recommendations. Medical staff can review this report, which provides a concise overview of the patient's condition, on a connected tablet. The report can be edited and validated by the medical staff, and forms the basis for clinical reasoning and final documentation.

In this study, Ada was exclusively used as a symptom checker to obtain structured symptom information. No proprietary scoring or diagnostic algorithms from Ada were evaluated, and all further processing (triage category assignment, suspected diagnosis, report generation) was performed by the locally running backend. Ada Health had no involvement in study design, data handling, analysis, or interpretation. Ada outputs themselves were not modified *post hoc* by staff; only the final system-generated reports could be reviewed and edited by clinicians as a part of routine validation.

**Figure 1:**
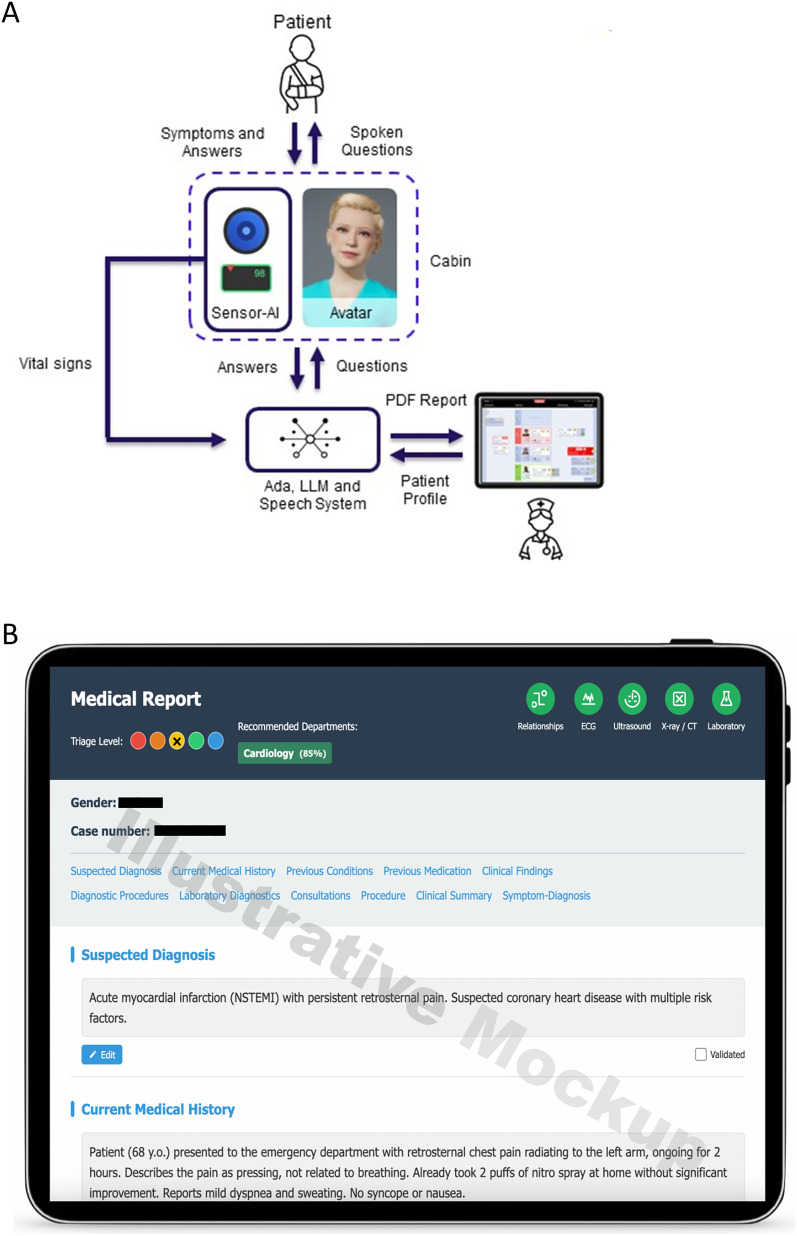
**(A)** Schematic structure of the modular AI platform. The patient interacts via spoken input with an avatar inside a diagnostic cabin equipped with sensors and a monitor. Vital signs and verbal responses are processed by a speech system and a large language model, generating a PDF report and patient profile for clinical review. Created using IconifyXR. *(B)* Illustrative mockup of a structured digital medical report interface (concept design generated with Claude Sonnet, Anthropic). The report includes organized clinical information such as suspected diagnosis, current medical history, as well as additional sections like previous conditions, procedures, and a final clinical summary. The interface also features triage level indicators, a department recommendation (Cardiology, 85%), and diagnostic tool icons (ECG, Ultrasound, x-ray/CT, Laboratory), which are part of the illustrative concept and not outputs of this study. Generated using Claude Sonnet by Anthropic (v3.7, San Francisco, CA, USA).

A schematic overview of the system architecture is provided in Figure 1A. Figure 1B presents an illustrative mockup (concept design generated with Claude Sonnet, Anthropic) of a structured medical report, created for illustrative purposes only. An image of the platform is provided in Supplementary Figure S1.

### 2.2 Participants and study design

In April 2025, ambulatory patients presenting to the ED of Marburg University Hospital were recruited for the study. Patients arriving by ambulance were excluded, as walk-in patients are the target group of the system. All participants initially underwent standard triage according to the German version of the Manchester Triage System (MTS). The MTS categorizes urgency into five levels: red (immediate treatment), orange (within 10 min), yellow (within 30 min), green (within 90 min), and blue (within 120 min) (33, 34). Only patients categorized as green or blue were included, as low-acuity patients comprise the main demographic to be processed by the AI-supported system. Eligibility criteria further required an age of ≥18 years, sufficient proficiency in German to interact with the avatar and adequate cognitive capacity to understand and follow instructions. Patients with acute cognitive impairment or insufficient German language proficiency were excluded. Digital literacy was not explicitly assessed, but none of the participants reported difficulties interacting with the system.

Following initial triage, eligible patients were directly approached by study staff in the ED waiting area and informed about the study. Participation was voluntary, and no financial or material incentives were offered. To recruit 20 participants, a total of 25 patients were approached; 5 declined participation, stated that they were not interested in taking part. After providing informed consent, participants received a brief technical introduction to the system. They were then connected to a monitor for continuous recording of oxygen saturation, respiratory rate, heart rate (via pulse oximetry), and body temperature. This was followed by a structured, dialog-based complete assessment using the AI platform, which included medical history-taking, generation of a suspected diagnosis, and automatic medical report creation. During the assessment, patients did not receive written prompts but interacted directly with the avatar. The avatar first posed standardized safety questions to exclude MTS red/orange criteria and, if these were ruled out, invited patients to freely describe their complaints.

**Figure 2:**
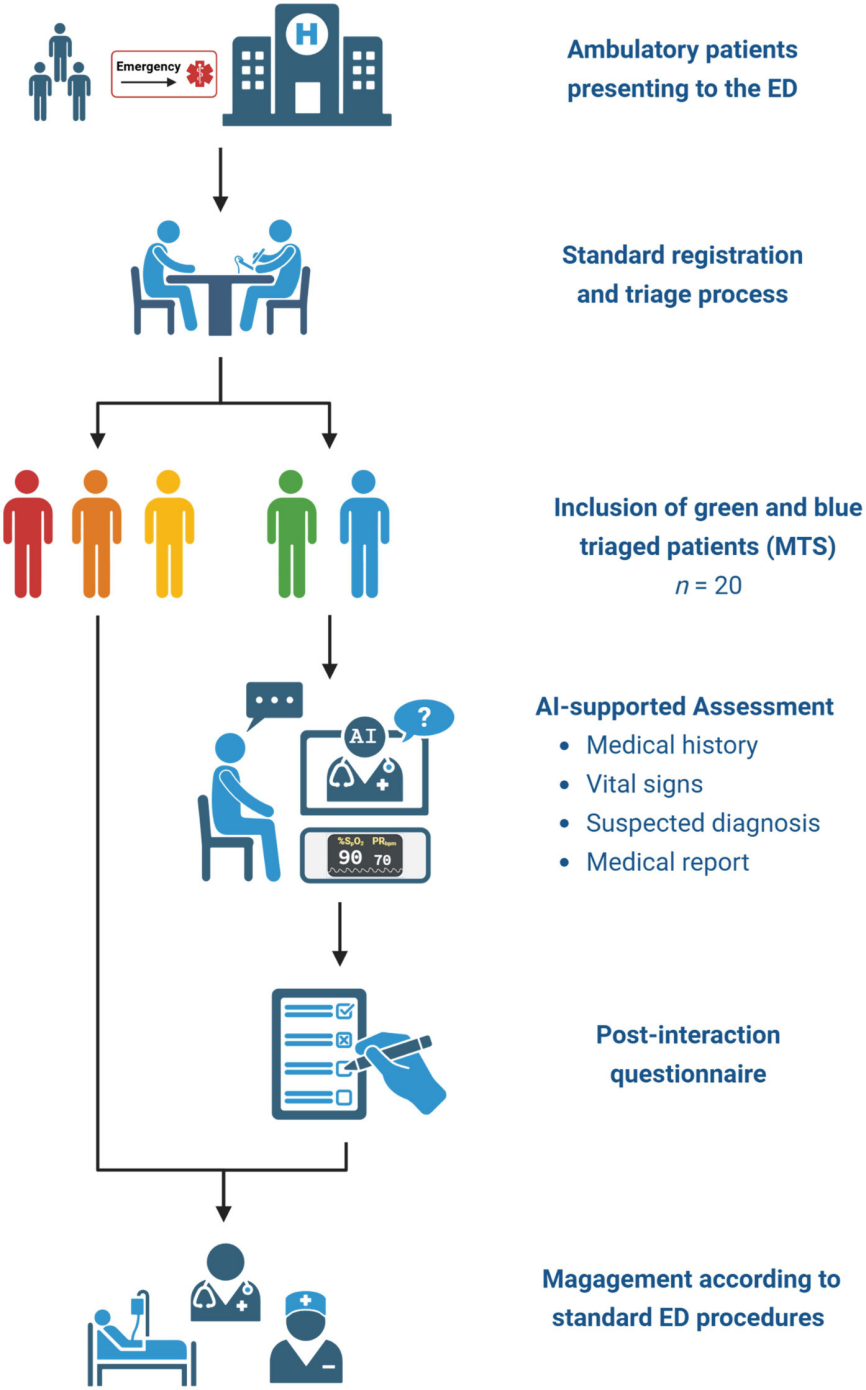
Flowchart of the study design. Ambulatory patients presenting to the emergency department (ED) were registered and triaged according to standard procedures. Patients categorized as green or blue based on the Manchester Triage System (*n* = 20) were included and underwent AI-supported assessment encompassing medical history, vital signs, and generation of a suspected diagnosis and medical report. Following the assessment, participants completed a post-interaction questionnaire. Clinical management proceeded according to standard ED protocols. Created using Biorender, licensed under Academic License.

Upon completion of the AI-supported assessment, participants were asked to complete a set of questionnaires (see Section 2.4). Subsequent clinical management proceeded in accordance with standard ED protocols. An overview of the study design is presented in Figure 2 and an exemplary setting is illustrated in Supplementary Figure S1.

The sample size for this exploratory study was set at 20 participants. This decision was made in consultation with the local ethics committee and aligns with established recommendations for feasibility and pilot studies. Julious proposed a “rule of 12 per group” as a pragmatic guideline to ensure stable estimates of variability in pilot contexts (35), while Hertzog emphasized that small sample sizes of around 20–30 are generally sufficient to assess feasibility objectives, and refine study procedures (36). In line with these principles, our study aimed to provide initial evidence of feasibility.

### 2.3 Consent to participate and ethics approval

As this was a pilot study, the number of participants was limited to 20 in consultation with the ethics committee of Marburg University Hospital. Written informed consent was obtained from all participants. The study was approved by the ethics committee of the University of Marburg (File Number “24-283 BO”). All procedures were conducted in accordance with the Declaration of Helsinki and complied with local regulations governing human subject research.

### 2.4 Questionnaire design

**Figure 3:**
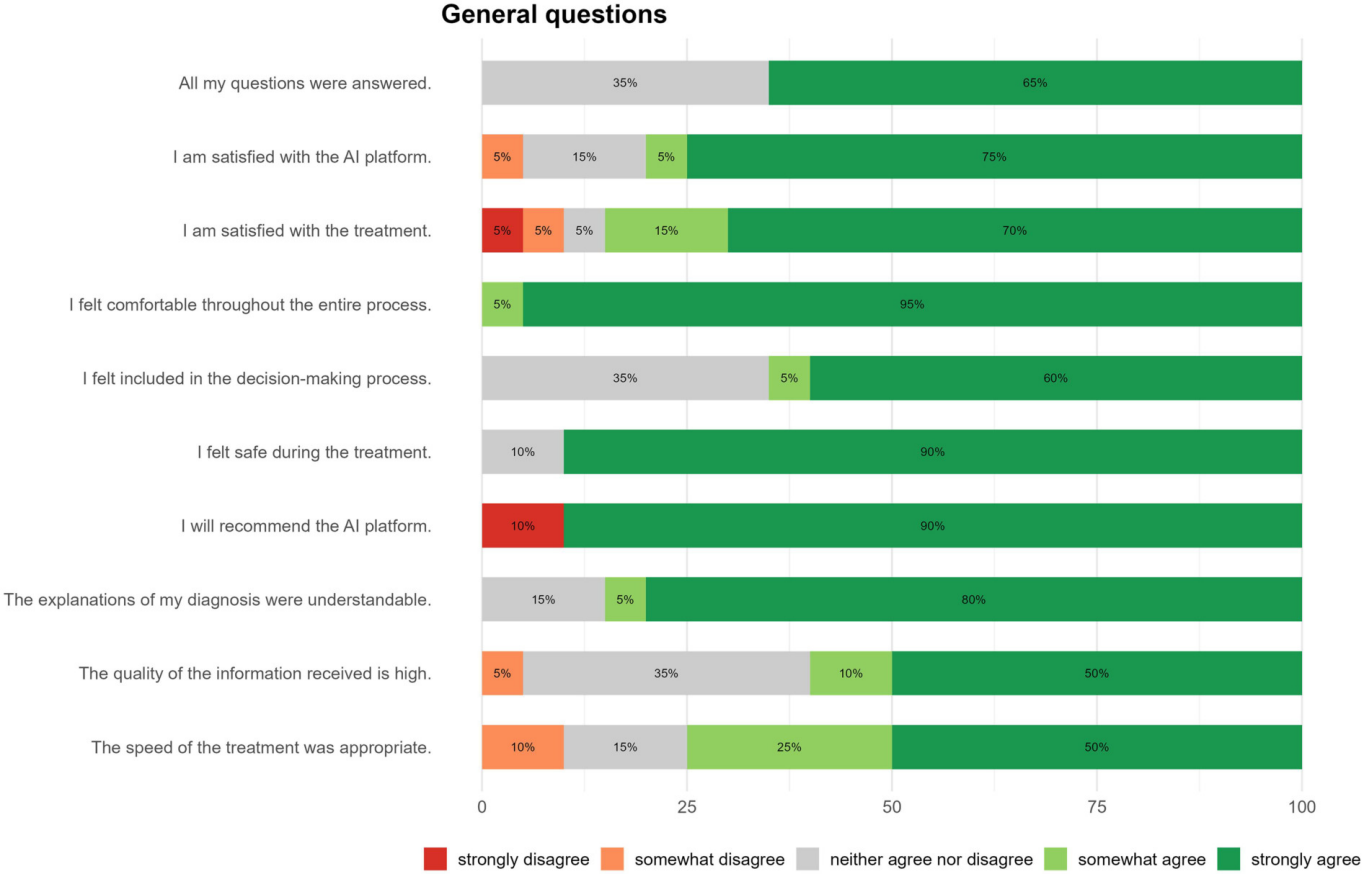
Overall evaluation of the AI platform. Patient feedback, collected using a five-point Likert scale, indicates high levels of satisfaction, comfort, and perceived safety with the AI system. Most participants expressed willingness to recommend the platform to others, while a smaller portion remained neutral regarding involvement in decision-making and the amount of information received.

In addition to demographic data (age and gender), further variables were collected, including medical specialty department, suspected diagnosis, triage category, and the duration of human–machine interaction. The survey comprised 39 items rated on a five-point Likert scale (1 = strongly disagree, 2 = somewhat disagree, 3 = neutral, 4 = somewhat agree, 5 = strongly agree), distributed across three separate questionnaires. The first questionnaire included ten self-developed items (see Figure 3). The second consisted of the validated System Usability Scale (SUS), containing ten items (37). The third included the validated Trust in Automation (TiA) questionnaire with 19 items (38).

The self-developed items were designed by the study team to capture aspects not fully addressed by the validated instruments, such as patients' perceived involvement in decision-making and the clarity of information provided. Item development was informed by prior work on patient satisfaction and usability in digital health but was not formally validated; they should therefore be regarded as exploratory and primarily intended to generate hypotheses and provide system-specific feedback for platform improvement. The full wording of all survey instruments (self-developed items, SUS and TiA) is provided in the Supplementary Material in both German (as administered) and English (Supplementary Tables S2–S4).

### 2.5 Data analysis and graphical illustration

Survey responses were manually digitized and entered into a Microsoft Excel spreadsheet (version 16, Redmond, WA, USA) for subsequent data analysis. Statistical evaluations and generation of visualizations were performed using R (version 4.5.0; Vienna, Austria) and RStudio (version 2024.12.1; Boston, MA, USA). To minimize transcription errors, a double-entry procedure was applied: each dataset was cross-checked by two researchers for consistency prior to statistical analysis in R. The anonymized dataset and the R analysis script are openly available (see Data Availability Statement).

Descriptive statistics included means, standard deviations (SD), medians, and 95% confidence intervals (CI) to characterize central tendency and data dispersion. Data are reported as mean ± SD and 95% CI, where applicable.

The SUS was analyzed according to standard procedures (37). The ten items were rated on a 5-point Likert scale (1 = strongly disagree to 5 = strongly agree). Negatively worded items (2, 4, 6, 8, 10) were reverse-coded. The overall SUS score was computed as the sum of all item scores multiplied by 2.5, yielding a range from 0 to 100. The TiA questionnaire was administered in its validated 19-item version (38), covering six subscales: Reliability and Competence items (items F1, F6, F10, F13, F15, F19), Understandability and Predictability (items F2, F7, F11, F16), Familiarity (items F3, F17), Intention of Developers (items F4, F8), Propensity to Trust (items F5, F12, F18), and General Trust (items F9, F14). Negatively worded items (items F5, F7, F10, F15, F16) were reverse-coded, and subscale means were computed accordingly. Cronbach's *α* was calculated for the 19-item total scale. For both instruments, Likert anchors matched the official instrument guidance exactly, and no missing data occurred.

Figure 1A was created using IconifyXR [2025, (39)]; Figure 1B was generated using Claude Sonnet by Anthropic (version 3.7, San Francisco, CA, USA) as a conceptual mockup for illustrative purposes. It does not represent the live or deployed user interface of the system. The study flowchart (Figure 2) was designed using BioRender (Toronto, Canada).

## 3 Results

### 3.1 Baseline characteristics of study participants

A total of 20 patients participated in this pilot study, comprising 14 women (70%) and 6 men (30%). The mean age was 45.1 years (SD ± 18.8), ranging from 18 to 86 years. The average interaction time was 10.6 min (SD ± 1.9). Interaction time refers to the duration of the AI-guided assessment (dialogue and data collection) and does not include rooming or sensor connection. Most patients (*n* = 14; 70%) were treated in the trauma surgery department. Three participants (15%) presented with neurological conditions, two (10%) with internal medicine issues, and one (5%) with a dermatological condition. The most frequent diagnoses involved extremity injuries, followed by non-traumatic orthopedic complaints. The majority of patients (*n* = 18; 90%) received a green triage classification. Baseline characteristics are summarized in Table 1.

**Table 1:** Baseline characteristics of study participants.

Sex	Number (%)
Female	14 (70%)
Male	6 (30%)
Age	Number (%)
Mean (±standard deviation)	45.1 (±18.8); 95% CI [36.0–54.1]
Min < Median < Max	18 < 42.5 < 86
Human-machine interaction time	Minutes
Mean (±standard deviation)	10.6 (±1.9); 95% CI [9.6–11.5]
Medical department	Number (%)
Trauma surgery	14 (70%)
Neurology	3 (15%)
Internal Medicine	2 (10%)
Dermatology	1 (5%)
Suspected diagnosis	Number (%)
Lower extremity trauma	7 (35%)
Upper extremity trauma	3 (15%)
Orthopedic complaints	3 (15%)
Spinal trauma	1 (5%)
Headache	1 (5%)
Dizziness	1 (5%)
Paresthesia	1 (5%)
Nausea	1 (5%)
Unspecified upper abdominal pain	1 (5%)
Herpes zoster	1 (5%)
Triage category	Number (%)
Green	18 (90%)
Blue	2 (10%)

Overview of patient characteristics and interaction metrics, including age, sex, triage category, medical department, suspected diagnoses, and duration of human-machine interaction. Interaction time refers to the duration of the AI-guided assessment (dialogue and data collection) and does not include rooming or sensor connection.

### 3.2 General evaluation results

Patient perceptions were assessed using a set of ten self-developed items (see Figure 3), addressing key aspects of usability, experience, and communication. Participants reported a high level of approval in key user experience areas. At least 80% of participants (*n* = 16) selected the most positive survey response, when asked to rate perceived safety, ease of use, overall satisfaction, and willingness to recommend the system. However, two participants explicitly disagreed with the statement about recommending the platform. Regarding the statement “The speed of the treatment was appropriate”, 75% (*n* = 15) of participants agreed strongly or somewhat agreed. When asked whether their questions were answered and whether they felt included in clinical decisions, 65% (*n* = 13) expressed agreement (either strongly or somewhat). Similarly, only 12 patients (60%) agreed that the information they received was of high quality (Figure 3).

### 3.3 Systems' usability

**Figure 4:**
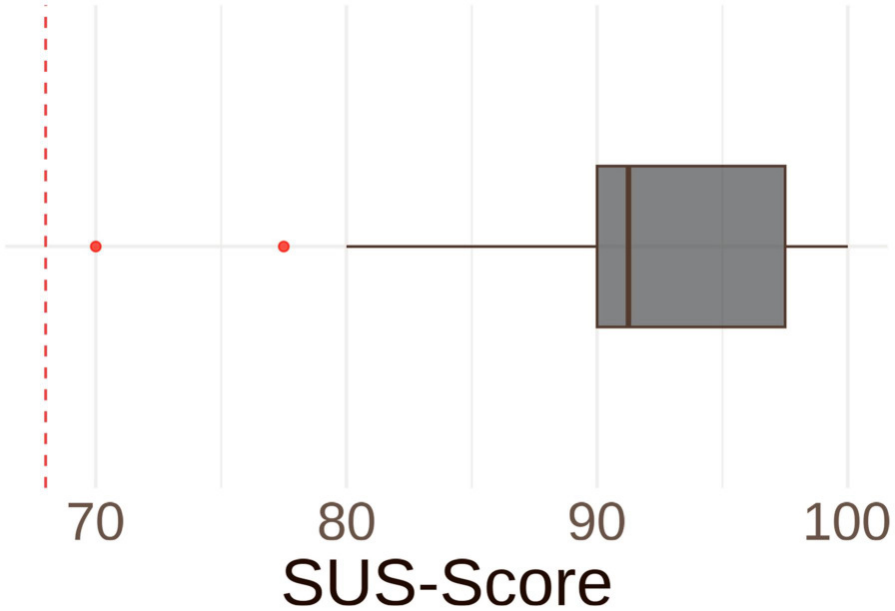
System usability scale (SUS)-score. Boxplot illustrating overall SUS-score. An SUS-score of 68 (red lined benchmark) or more indicates good usability, while a score above 80.3 points indicates excellent usability. All participants achieved at least the benchmark score of 68. With a mean of 90.6 points (SD ± 7.9; IQR: 90.0–97.5), the AI platform achieved an excellent usability.

Usability was assessed using the validated SUS. Internal consistency in our sample was low (Cronbach's *α* = 0.44), which is expected given the very limited variance in responses: negatively worded items clustered at the minimum, while positively worded items clustered at the maximum (see Supplementary Table S5). This pattern reflects the uniformly high ratings of ease of use. All participants strongly agreed that the platform is easy to use. Accordingly, nobody felt that the product was unnecessarily complex. In the overall assessment, a SUS score of 68 or more indicates good usability, while a score above 80.3 points indicates excellent usability, following the standard adjective rating bands (37, 40). None of the patients gave a score below the benchmark of 68. With an average SUS score of 90.6 (SD ± 7.9; IQR 90.0–97.5), the AI-based platform achieved an excellent usability (Figure 4 and Table 2).

**Table 2:** Summary of questionnaire results (system usability scale overall score and trust in automation subscales).

System Usability Scale (SUS)	Mean (±SD)
Overall score	90.6 (7.9)
Trust in automation	
Reliability and competence	3.62 (±0.41)
Understandability and predictability	4.12 (±0.81)
Familiarity	1.62 (±1.32)
Intention of developers	4.40 (±0.74)
Propensity to trust	3.30 (±1.06)
General trust	3.92 (±0.96)

Values are presented as mean ± standard deviation (SD); *n* = 20. Item-level descriptive statistics are provided in the Supplementary Tables S5 and S6.

### 3.4 Trust in automation

To provide a more nuanced picture, we report results for the subdimensions of the TiA questionnaire. The TiA reflects three dimensions of perceived trustworthiness (41, 42): performance (i.e., perceived competence and reliability of the system), process (i.e., understandability and predictability), purpose (i.e., developers' intentions). All dimensions were rated on a five-point Likert scale. Negatively worded items were reverse-scored so that higher scores consistently represent more positive evaluations, with 5 indicating the most favorable possible rating. Internal consistency of the TiA questionnaire in our sample was acceptable (Cronbach's *α* = 0.76). The performance dimension was rated neutral to slightly positive, with a mean score of 3.62 (SD ± 0.41). Developers' intentions were perceived positively, with a mean score of 4.40 (SD ± 0.74), indicating that the system was viewed as ethically well-intentioned. The perceived understandability and predictability of the system were rated positively, with an average score of 4.12 (SD ± 0.81). Ratings for the general trust in the automated system were average to slightly positive (*M* = 3.92; SD ± 0.96).

**Figure 5:**
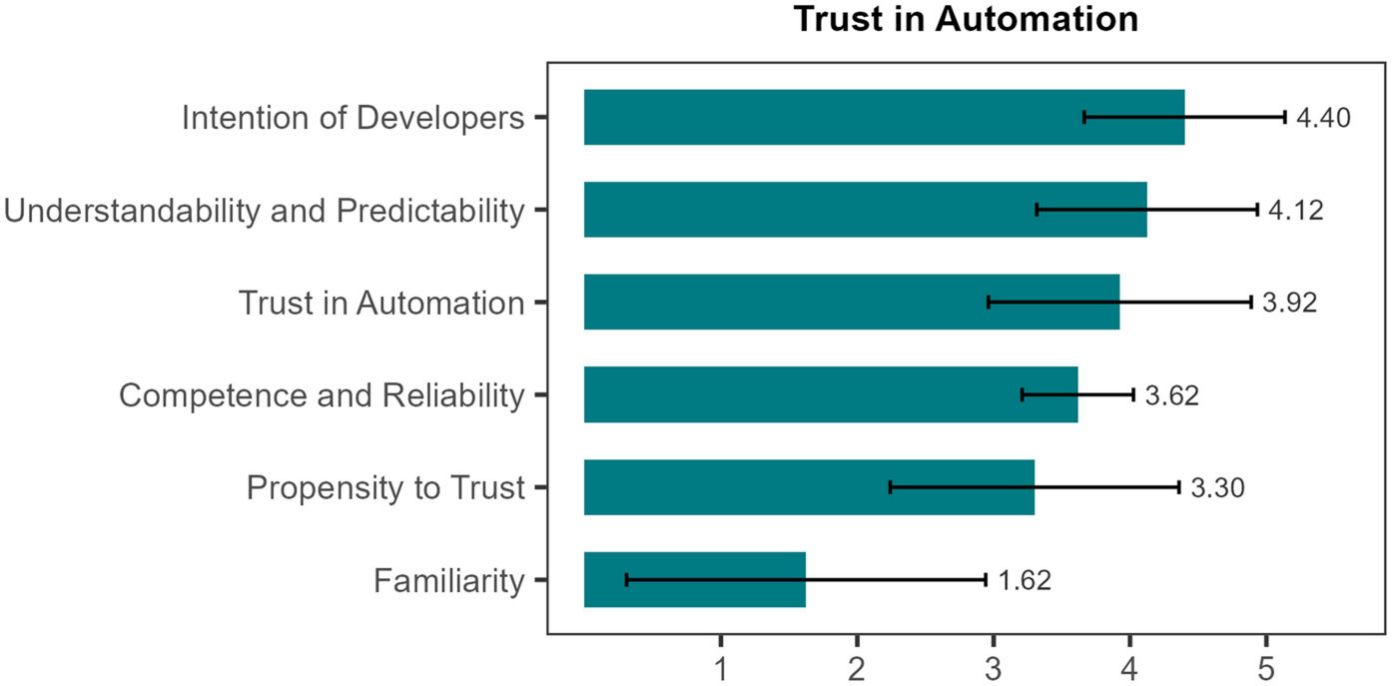
Trust in automation. Mean scores with standard deviations (SDs) are shown for six subscales of Trust in Automation, measured on a 5-point Likert scale (1 = strongly disagree, 2 = somewhat disagree, 3 = neutral, 4 = somewhat agree, 5 = strongly agree). With a mean of 4.40 (±0.74) points participants rated intention of developers highest, followed by understandability and predictability (mean 4.12 ± 0.81), while familiarity received the lowest ratings (mean 1.62 ± 1.32), indicating limited prior exposure to the system.

Beyond system-related assessment, the TiA also captures participants' general tendency to trust and rely on automated systems (i.e., propensity to trust), as well as their familiarity with similar systems. The mean score for trust propensity was 3.30 (SD ± 1.06), indicating a balanced attitude toward automation. Familiarity with similar systems was low (*M* = 1.62; SD ± 1.32), indicating little to no previous experience. However, the relatively high standard deviations for both measures indicate considerable individual variability in trust propensity and prior experience (Figure 5 and Table 2). Item-level descriptives, including floor and ceiling distributions, are provided in Supplementary Table S6.

## 4 Discussion

In this pilot study, we deployed a multimodal AI-based prototype platform in an ED setting for the first time. The results demonstrate a high level of patient satisfaction, excellent usability and a high level of trust in the developers of the technology. This underlines the fundamental acceptance and potential of such innovative systems in the clinical emergency setting, even among patients with limited prior exposure to such technologies. Our initial implementation represents a foundational step toward broader clinical integration, which may offer long-term benefits in addressing ED overcrowding.

### 4.1 Potential benefits for reducing ED overcrowding

#### 4.1.1 Input factors

By standardizing and automating medical history-taking, vital signs recording, and AI-supported preliminary assessment, the platform may provide valuable information while patients are still in the waiting area. This perspective aligns with recent systematic reviews, which consistently identify vital signs as dominant predictors and emphasize the added value of integrating structured symptom information and unstructured clinical text into triage models (21, 22). In principle, this could support more consistent triage and earlier patient management, though this was not assessed in the present study. Once diagnostic accuracy has been validated in larger trials, the system might also recommend outpatient follow-up instead of ED presentation in selected cases. Furthermore, the automated recording of vital signs could potentially facilitate early detection of critical conditions such as sepsis, which remains an important future outcome to investigate. Early identification of sepsis is particularly relevant in overcrowded EDs, where increased patient volume is linked to higher sepsis-related mortality (43). An additional benefit of this platform could be the potential reduction in patient waiting times, which is known to improve overall patient satisfaction (44).

#### 4.1.2 Throughput factors

During the ED workflow, the AI platform may in future reduce administrative workload by automating documentation and generating structured medical reports without further straining the limited human resources. This may allow clinicians to devote more time to direct patient care. Such tools may prove especially valuable in light of ongoing staff shortages (45, 46) and increasing documentation demands (47, 48).

As a technical solution, the system is unaffected by shift schedules and sick leave, and can be used around the clock. Standardized triage-supporting recordings may help reduce variability, particularly among less experienced staff. They could also minimize typical sources of error that can arise due to stress, fatigue or frequent staff changes. Standardization has been shown to improve reliability in clinical practice (49–51). Additionally, the system's action recommendations may help to ensure that necessary diagnostics are initiated earlier, thus shortening the time from presentation to clinical decision-making.

#### 4.1.3 Output factors

Early, structured information gathering could, in theory, indirectly support resource allocation by improving estimates of treatment needs and likelihood of admission. More accurate prognoses regarding treatment duration and urgency may help streamline discharge and transfer processes over the medium to long term. These potential benefits remain speculative and were not examined in this pilot study. Recent reviews likewise underline that while AI models show promise, prospective real-world validation, particularly with operationally relevant outcome measures, remains scarce (21, 26, 28).

### 4.2 Challenges and technical limitations

One major limitation is the relatively long average duration of the interaction time with the system (10.6 min). For context, conventional triage performed by clinical staff typically requires around 4.0 min, including assessment of vital signs (52, 53). This could be problematic in time-critical emergencies such as myocardial infarction or sepsis. Therefore, the current system is not suitable for high-urgency cases (MTS red or orange). These patients must still be identified and assessed by clinical staff. The platform is programmed to terminate the dialogue as soon as indicators of red or orange triage emerge and to notify clinical staff via tablet. However, such high-urgency cases are relatively rare among ambulatory ED presentations (54) and are not the cause of ED overcrowding. Instead, our system aims at reallocating resources to where staff has more time to attend to critical cases. Importantly, the platform's utility extends beyond triage alone: it collects patient history, generates a structured report, and provides clinical recommendations, making it a comprehensive tool for initial assessment.

### 4.3 Workforce, organizational, cost, and ethical considerations

In addition to technical feasibility, broader workforce, organizational, economic, and ethical dimensions must be considered when evaluating the potential of AI-based platforms in EDs. Integration of AI systems may alter existing clinical workflows and work design, with potential implications for staff roles and competencies (55). Infrastructural dependencies have also been identified as critical, together with ethical, data security, and algorithmic fairness challenges (56). Cost and resource implications are equally important. While empirical evidence on procurement and maintenance remains scarce, recent modeling studies in ED settings demonstrate that AI-supported echocardiography or radiograph review can influence budget impact and cost savings (57, 58). From an ethical and legal perspective, issues of accountability in clinical decision-making remain unresolved, underlining the importance of transparency and trust-building (59). In line with this, Pinero de Plaza et al. recently introduced a participatory evaluation framework in emergency cardiac care (PROLIFERATE_AI), which emphasizes stakeholder involvement, usability, and trust-related dimensions as key factors for safe and equitable AI deployment (60).

### 4.4 Patient participation and potential for improvement

The study also revealed a desire among participants for greater involvement in decision-making. Suggested improvements included clearer explanations of suspected diagnoses and reasoning, as well as a concluding summary at the end of the interaction. While these measures could strengthen acceptance and trust, they would also extend the length of the interaction. One possible solution would be to delegate some of the explanatory elements to the healthcare professional, depending on the situation.

### 4.5 Technological context of acceptance

Technology was used by an inexperienced sample (with 80% indicating unfamiliarity or low prior experience according to the TiA questionnaire's familiarity subscale, reflected by a mean score of 1.62), a high level of acceptance was recorded. However, two patients (10%) completely rejected the system, possibly due to skepticism towards new technologies in the general population (61, 62). Since participation in this study was voluntary, the population-wide rejection rate may be higher if system use were mandatory. Greater availability and visibility of such technologies in clinical settings may help normalize their use and improve long-term acceptance.

### 4.6 Alignment with recent systematic reviews

Systematic reviews have underlined the central role of vital signs, the added value of unstructured free-text-based information, and feasibility constraints such as EHR integration, interpretability, and clinician acceptance. Our platform reflects some of these themes by incorporating vital signs and combining structured symptom intake with LLM-based processing, which allows elements of narrative data to be considered. At the same time, challenges around EHR integration, interpretability, and clinician acceptance were not addressed in this pilot and remain important directions for future work.

### 4.7 Limitations

While the implementation was feasible, well-received, and associated with high patient trust, this study has several limitations. As an exploratory pilot study, it included a small sample size (N = 20) and followed a monocentric design. The focus was limited to feasibility and patient-reported acceptance. We did not assess patient-flow, diagnostic accuracy, or operational outcomes such as time-to-triage, length of stay, or left-without-being-seen rates. Accordingly, any implications regarding ED overcrowding, throughput, or resource allocation remain speculative and should be interpreted only as potential directions for future work. In addition, the restriction to non-urgent (MTS green/blue) walk-in patients, most of whom presented with trauma or orthopedic conditions, introduces a potential spectrum bias and limits the generalizability of our findings to higher-acuity patient groups. In addition, participation was voluntary, and 5 of 25 eligible patients declined to take part, mainly due to lack of interest. As such, the study population may be biased toward individuals more open to novel technologies, which could have positively influenced satisfaction and trust ratings. The brief data collection period presents another limitation.

Future studies should therefore be multicentric, extend longer periods, and include more heterogeneous cohorts, particularly older adults and patients with medium or high acuity, to directly assess diagnostic accuracy, patient-flow indicators, and system-level effects. Such designs directly respond to the recommendations of recent integrative reviews, which call for multicenter prospective validation, seamless EHR integration, and transparent reporting of operational outcomes to establish the real-world impact of AI-assisted triage (21, 26, 28). In line with these recommendations, future evaluations should incorporate concrete operational metrics such as time-to-initial-assessment, left-without-being-seen-rates, admission and intensive care unit prediction calibration, under- and over-triage rates, and staff documentation time.

## 5 Conclusion

In this usability-focused pilot study, we found that a modular and multimodal AI-based platform can be deployed in an ED setting and was well accepted by a small sample of patients. The system achieved excellent usability, with a high average SUS score, and participants reported a generally positive perception and trust, particularly regarding developers' intentions. The structured collection of clinical information and the automatic generation of medical reports represent technical functions that warrant further evaluation regarding their potential contribution to standardization and efficiency in emergency care workflows. Future research should focus on validating diagnostic performance, evaluating integration into clinical decision-making processes, and assessing the platform's effectiveness in reducing ED overcrowding, including operational outcomes such as time-to-initial-assessment, triage accuracy, and documentation efficiency.

## Data Availability

The datasets presented in this study can be found in online repositories. The names of the repository/repositories and accession number(s) can be found below: Zenodo. https://doi.org/10.5281/zenodo.17091901.
