# Supplementary material for: Feasibility of a multimodal AI-based clinical assessment platform in emergency care: an exploratory pilot study
Source: Front Digit Health. 2025 Oct 3;7:1657583. doi: 10.3389/fdgth.2025.1657583 (PMC12533282; doi:10.3389/fdgth.2025.1657583)
Supplement: Supplementary file 1 [file Datasheet1.docx]

Supplementary Material

***Feasibility of a Multimodal AI-Based Clinical Assessment Platform in Emergency Care: An Exploratory Pilot Study***

**
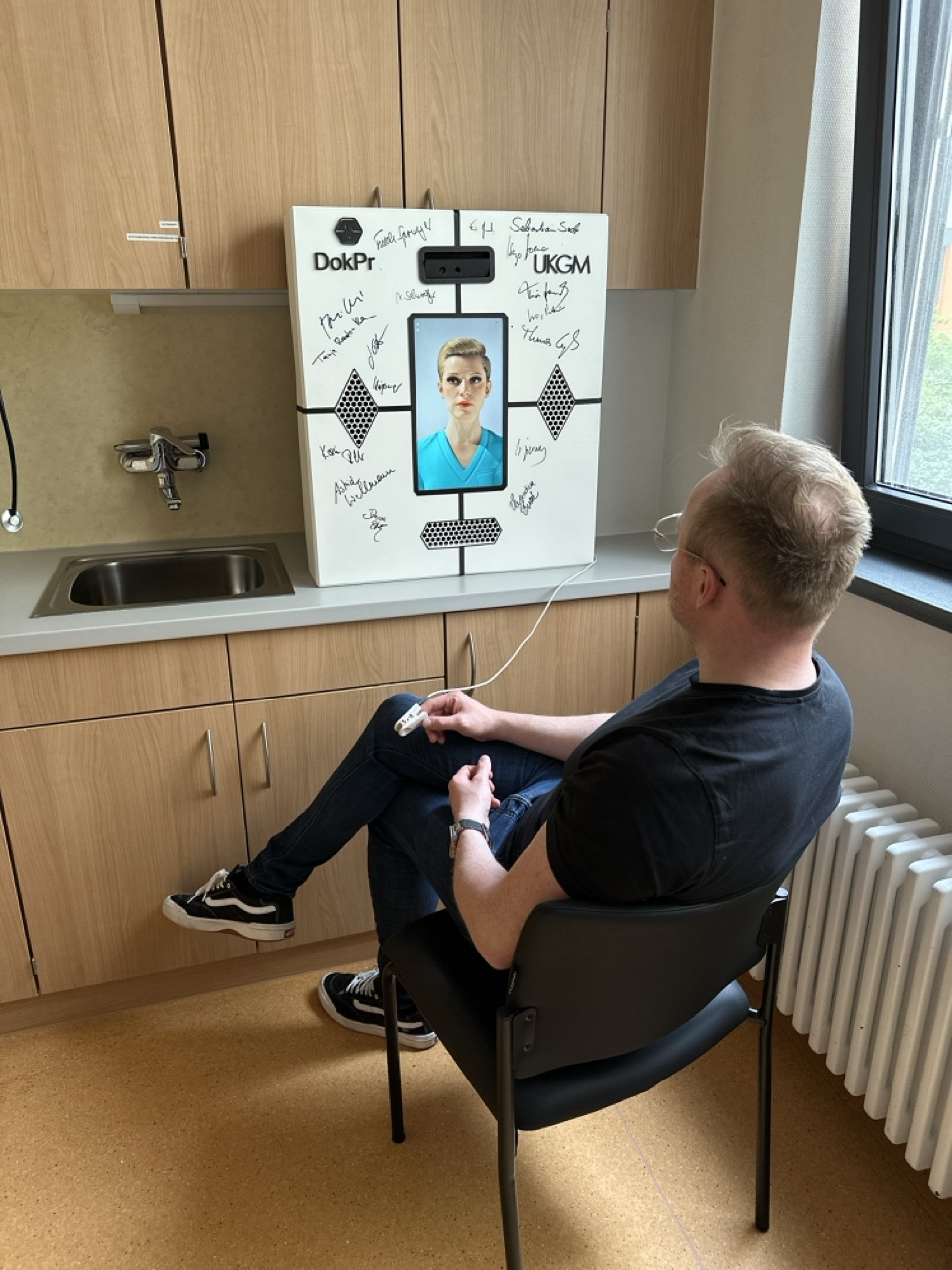
**

**Supplementary Figure 1 | Snapshot of the study setting.** A participant interacts with the modular AI platform in a clinical room setting. The setup includes a wall-mounted device featuring a screen-based medical avatar and integrated sensors, enabling contactless history taking and physiological data acquisition. Note: Photograph was taken by the authors and is used with permission.

**Supplementary Table S1. Model card and safety guardrails for the Large Language Model (LLM) used in this study.**

| **Aspect** | **Information** |
| --- | --- |
| Model name | Mistral Small 3 (mistral-small:24b) |
| Developer | Mistral AI, Paris, France |
| Release year | 2024 |
| Access | Locally deployed; no external API |
| Intended use in this study | Processing structured symptom and vital sign data to generate triage suggestions, suspected diagnosis, and draft medical reports |
| Training data | Mistral Small 3 was released under the Apache 2.0 license, trained with multilingual text corpora and instruction tuning for broad generative AI tasks (Mistral AI, 2025). Precise details of the training datasets are described in the model documentation. |
| Safety guardrails implemented | 1. Local deployment to ensure data privacy and avoid transmission of personal health information. 2. Automatic termination of dialogue if MTS red/orange indicators detected, with immediate alert to clinical staff. 3. Permanent availability of a board-certified emergency physician during all study procedures (ethics requirement). |
| Post-processing | No modification of Ada outputs; LLM-generated drafts were available for staff review but not altered post hoc in this study. |
| Full model card | https://mistral.ai/news/mistral-small-3 (last accessed September 12, 2025) |

**Supplementary Table S2. System Usability Scale (SUS) items (German version used in this study, with English equivalents; (Brooke 1995). Note:** All items were rated on a 5-point Likert scale (1 = strongly disagree, 5 = strongly agree). Items 2, 4, 6, 8, and 10 were reverse-coded before scoring. Total SUS scores were calculated by summing item contributions and multiplying by 2.5 (yielding a 0–100 scale).

| **Item** | **German wording (administered)** | **English wording** | **Scoring** |
| --- | --- | --- | --- |
| F1 | Ich denke, dass ich das System gerne häufig benutzen würde. | I think that I would like to use this system frequently. | **+** |
| F2 | Ich fand das System unnötig komplex. | I think that I would like to use this system frequently. | **-** |
| F3 | Ich fand das System einfach zu benutzen. | I thought the system was easy to use. | **+** |
| F4 | Ich glaube, ich würde die Hilfe einer technisch versierten Person benötigen, um das System benutzen zu können. | I think that I would need the support of a technical person to be able to use this system. | **-** |
| F5 | Ich fand, die verschiedenen Funktionen in diesem System waren gut integriert. | \| I found the various functions in this system were well integrated. \| \| --- \|  \|  \| \| --- \| | **+** |
| F6 | Ich denke, das System enthielt zu viele Inkonsistenzen. | I thought there was too much inconsistency in this system. | **-** |
| F7 | Ich kann mir vorstellen, dass die meisten Menschen den Umgang mit diesem System sehr schnell lernen. | I would imagine that most people would learn to use this system very quickly. | **+** |
| F8 | Ich fand das System sehr umständlich zu nutzen. | I would imagine that most people would learn to use this system very quickly. | **-** |
| F9 | Ich fühlte mich bei der Benutzung des Systems sehr sicher. | I felt very confident using the system. | **+** |
| F10 | Ich musste eine Menge lernen, bevor ich anfangen konnte das System zu verwenden. | I felt very confident using the system. | **-** |

**Supplementary Table S3. Trust in Automation (TiA) questionnaire items (German version used in this study, with English equivalents; (Körber 2019). Note:** All items were rated on a 5-point Likert scale (1 = strongly disagree, 5 = strongly agree). Negatively worded items (F5, F7, F10, F15, F16) were reverse-coded prior to analysis. Subscale scores were computed as the mean of their respective items.

| **Item** | **German wording (administered)** | **English wording** | **Scoring** |
| --- | --- | --- | --- |
| F1 | Das System ist imstande, Situationen richtig einzuschätzen. | The system is capable of interpreting situations correctly. | **+** |
| F2 | Mir war durchgehend klar, in welchem Zustand sich das System befindet. | The system state was always clear to me. | **+** |
| F3 | Ich kenne bereits ähnliche Systeme. | I already know similar systems. | **+** |
| F4 | Die Entwickler sind vertrauenswürdig. | The developers are trustworthy. | **+** |
| F5 | Bei unbekannten automatisierten Systemen sollte man eher vorsichtig sein. | \| One should be careful with unfamiliar automated systems. \| \| --- \|  \|  \| \| --- \| | **-** |
| F6 | Das System arbeitet zuverlässig. | The system works reliably. | **+** |
| F7 | Das System reagiert unvorhersehbar. | The system reacts unpredictably. | **-** |
| F8 | Die Entwickler nehmen mein Wohlergehen ernst. | The developers take my well-being seriously. | **+** |
| F9 | Ich vertraue dem System. | I trust the system. | **+** |
| F10 | Ein Ausfall des Systems ist wahrscheinlich. | A system malfunction is likely. | **-** |
| F11 | Ich konnte nachvollziehen, warum etwas passiert ist. | I was able to understand why things happened. | **+** |
| F12 | Ich vertraue einem System eher, als dass ich ihm misstraue. | I rather trust a system than I mistrust it. | **+** |
| F13 | Das System kann wirklich komplizierte Aufgaben übernehmen. | The system is capable of taking over complicated tasks. | **+** |
| F14 | Ich kann mich auf das System verlassen. | I can rely on the system. | **+** |
| F15 | Das System könnte stellenweise einen Fehler machen. | The system might make sporadic errors. | **-** |
| F16 | Zu erkennen, was das System als nächstes macht, ist schwer. | It is difficult to identify what the system will do next. | **-** |
| F17 | Ich habe ähnliche Systeme bereits genutzt. | I have already used similar systems. | **+** |
| F18 | Automatisierte Systeme funktionieren generell gut. | Automated systems generally work well. | **+** |
| F19 | Ich bin überzeugt von den Fähigkeiten des Systems. | I am confident about the system’s capabilities | **+** |

**Supplementary Table S4. Self-developed questionnaire items (German version used in this study, with English equivalents). Note:** All items were rated on a 5-point Likert scale (1 = strongly disagree, 5 = strongly agree).

| **Item** | **German wording (administered)** | **English wording** |
| --- | --- | --- |
| F1 | Die Geschwindigkeit der Behandlung war angemessen. | The speed of the treatment was appropriate. |
| F2 | Ich bin zufrieden mit der Behandlung. | I am satisfied with the treatment. |
| F3 | Ich habe mich während der Behandlung sicher gefühlt. | I felt safe during the treatment. |
| F4 | Die Erklärungen zu meiner Diagnose waren verständlich. | The explanations of my diagnosis were understandable. |
| F5 | Die Qualität der erhaltenen Informationen ist hoch. | \| The quality of the information I received is high. \| \| --- \|  \|  \| \| --- \| |
| F6 | Ich habe mich während des gesamten Prozesses wohl gefühlt. | I felt comfortable throughout the entire process |
| F7 | Meine Fragen wurden alle beantwortet. | All of my questions were answered. |
| F8 | Ich werde das System weiterempfehlen. | I would recommend the systems to others. |
| F9 | Ich fühlte mich in den Entscheidungsprozess mit eingebunden. | I felt included in the decision-making process. |
| F10 | Ich bin zufrieden mit dem System. | I am satisfied with the system. |

**Supplementary Table S5. Item-level descriptive statistics for the System Usability Scale (SUS, n = 20)**

| **Item** | **Mean** | **Standard deviation** | **Minimum** | **Maximum** | **Floor % (1)** | **Ceiling % (5)** |
| --- | --- | --- | --- | --- | --- | --- |
| 1 | 4.5 | 1.0 | 1 | 5 | 5 | 70 |
| 2 | 1.2 | 0.4 | 1 | 2 | 85 | 0 |
| 3 | 5.0 | 0 | 5 | 5 | 0 | 100 |
| 4 | 1.5 | 1.1 | 1 | 5 | 80 | 5 |
| 5 | 4.6 | 0.7 | 3 | 5 | 0 | 75 |
| 6 | 2.2 | 1.3 | 1 | 5 | 40 | 10 |
| 7 | 4.6 | 0.9 | 2 | 5 | 0 | 75 |
| 8 | 1.2 | 0.5 | 1 | 3 | 90 | 0 |
| 9 | 4.7 | 0.6 | 3 | 5 | 0 | 75 |
| 10 | 1.1 | 0.2 | 1 | 2 | 95 | 0 |

**Supplementary Table S6. Item-level descriptive statistics for the Trust in Automation (TiA, n = 20)**

| **Item** | **Mean** | **Standard deviation** | **Minimum** | **Maximum** | **Floor % (1)** | **Ceiling % (5)** |
| --- | --- | --- | --- | --- | --- | --- |
| 1 | 4.4 | 0.7 | 3 | 5 | 0 | 50 |
| 2 | 4.4 | 0.9 | 3 | 5 | 0 | 65 |
| 3 | 1.7 | 1.4 | 1 | 5 | 80 | 10 |
| 4 | 4.3 | 0.8 | 3 | 5 | 0 | 45 |
| 5 | 3.8 | 1.5 | 1 | 5 | 15 | 45 |
| 6 | 4.5 | 0.8 | 3 | 5 | 0 | 65 |
| 7 | 1.9 | 1.1 | 1 | 4 | 55 | 0 |
| 8 | 4.6 | 0.7 | 3 | 5 | 0 | 70 |
| 9 | 4.2 | 1.1 | 2 | 5 | 0 | 60 |
| 10 | 3.7 | 1.0 | 2 | 5 | 0 | 30 |
| 11 | 4.5 | 0.8 | 3 | 5 | 0 | 70 |
| 12 | 4.1 | 1.2 | 1 | 5 | 10 | 50 |
| 13 | 4.2 | 0.9 | 3 | 5 | 0 | 55 |
| 14 | 3.7 | 1.0 | 2 | 5 | 0 | 25 |
| 15 | 3.9 | 1.0 | 2 | 5 | 0 | 35 |
| 16 | 2.5 | 1.5 | 1 | 5 | 40 | 15 |
| 17 | 1.6 | 1.3 | 1 | 5 | 80 | 10 |
| 18 | 3.6 | 1.4 | 1 | 5 | 15 | 40 |
| 19 | 4.2 | 0.8 | 3 | 5 | 0 | 45 |

References

Brooke, John (1995): SUS: A quick and dirty usability scale. In: *Usability Eval. Ind.* 189.

Körber, Moritz (2019): Theoretical Considerations and Development of a Questionnaire to Measure Trust in Automation. In: Sebastiano Bagnara, Riccardo Tartaglia, Sara Albolino, Thomas Alexander und Yushi Fujita (Hg.): Proceedings of the 20th Congress of the International Ergonomics Association (IEA 2018). Cham, 2019. Cham: Springer International Publishing, S. 13–30.
